# Comparative Efficacy of Robotic-Assisted Versus Laparoscopic Resection for Colorectal Cancer: A Systematic Review and Meta-Analysis of Randomized Controlled Trials

**DOI:** 10.7759/cureus.102032

**Published:** 2026-01-21

**Authors:** Khalid B Mohammed, Mokhtar Kahin, Abdelhadi A Okasha, Raed R Alshagager, Abdullah M Almutawa, Yasir Alkhatib, Sarah F Alanazi, Fatimah Y Alfaraj, AlKhansa S Al Mustafa, Abdulrahman K Alshuaib, Khaled E Barakat

**Affiliations:** 1 Surgery, International Medical Center, Jeddah, SAU; 2 Surgery, Jordan University Hospital, Amman, JOR; 3 Surgery, Alexandria University, Alexandria, EGY; 4 Surgery, King Saud bin Abdulaziz University for Health Sciences, Jeddah, SAU; 5 Paediatric Surgery, Hull University Teaching Hospitals, Birmingham, GBR; 6 Medicine, Tabuk University, Tabuk, SAU; 7 Surgery, Imam Abdulrahman Bin Faisal University, Khobar, SAU; 8 Surgery, Al-Ribat University, Khartoum, SDN; 9 Surgery, Al Sabah Hospital, Kuwait City, KWT; 10 Surgery, Faculty of Medicine, Alexandria University, Alexandria, EGY

**Keywords:** colectomy, colorectal neoplasms, laparoscopy, meta-analysis, proctectomy, randomized controlled trial, robotic surgical procedures, treatment outcome

## Abstract

Minimally invasive surgery is the standard of care for colorectal cancer resection. Robotic-assisted surgery (RAS) offers technical advantages over conventional laparoscopic surgery (LACS), including superior visualization and dexterity, but its clinical superiority is debatable due to higher costs and longer operative times. Previous meta-analyses were limited by the inclusion of non-randomized studies. This systematic review and meta-analysis of randomized controlled trials (RCTs) aimed to provide a definitive comparison of the perioperative, functional, and oncological outcomes. A systematic search was conducted from January 2000 to December 2025. Only RCTs comparing RAS and LACS for curative-intent colorectal cancer resection were included. The primary outcomes were conversion to open surgery, operative time, and estimated blood loss. The secondary outcomes included length of hospital stay, complications (specifically Clavien-Dindo grade ≥III), and pathological metrics. The risk of bias (RoB) was assessed using the Cochrane RoB 2 tool. A meta-analysis was performed using a random-effects model. Fifteen RCTs representing thirteen unique RCTs involving 2,965 (1,487 RAS and 1,478 LACS) patients were included. RAS was associated with a significantly lower rate of conversion to open surgery (risk ratio (RR): 0.52; 95% CI: 0.35 to 0.78; p=0.001) and reduced hospital length of stay (weighted mean difference (WMD): -0.73 days; 95% CI: -1.28 to -0.19; p=0.009). Operative time was significantly longer in the RAS group (WMD: +39.26 min; p<0.001). RAS resulted in a lower rate of circumferential resection margin (CRM) positivity (RR: 0.61; 95% CI: 0.44 to 0.86; p=0.005), while lymph node harvest and overall complication rates were comparable between the groups. RAS demonstrates clear clinical benefits over conventional laparoscopy for colorectal cancer resection, specifically in reducing conversion rates, shortening hospital stay, and improving CRM status, which support the continued adoption of RAS, particularly for complex rectal cancer cases, despite the longer operative times. Future research should focus on long-term functional outcomes and their cost-effectiveness.

## Introduction and background

Colorectal cancer is a predominant cause of cancer-related morbidity and mortality, requiring surgical resection as the curative treatment for non-metastatic disease [1,2]. The surgical management of colorectal malignancies has undergone a shift from open laparotomy to minimally invasive surgery. Supported by high-quality evidence from landmark trials such as CLASICC, COLOR, and COREAN, conventional laparoscopic surgery (CLS) is established as the standard of care for early-stage and selected locally advanced cases, offering validated short-term benefits over open surgery, including reduced postoperative pain, shorter hospital stays, and faster recovery of bowel function, without compromising long-term oncological safety [3,4].

Laparoscopy has limitations that can impede surgical performance, particularly during complex procedures such as total mesorectal excision (TME) or complete mesocolic excision, as conventional laparoscopy is constrained by two-dimensional visualization, a limited range of instrument motion, the fulcrum effect of rigid instruments, and amplification of physiological tremors [4,5]. These technical challenges are exacerbated in patients with unfavorable anthropometry, such as those with obesity, a narrow male pelvis, or locally advanced tumors, increasing the risk of conversion to open surgery and compromising circumferential resection margins [2,6].

Robotic-assisted surgery (RAS) was introduced to mitigate the technical deficiencies of CLS, as the robotic platform offers stereoscopic high-definition 3D visualization, endowristed instruments with seven degrees of freedom, and superior ergonomics, facilitating precise dissection in confined anatomical spaces [5,7]. Although RAS has demonstrated feasibility and safety, its clinical superiority over laparoscopy is debated as RAS reduces conversion rates, intraoperative blood loss, and nerve injury, thereby improving functional outcomes [6]. Critics highlight higher operative costs and prolonged operative times without definitive evidence of improved long-term oncological survival [4,8].

Literature synthesizing these surgical approaches is confounded by the inclusion of non-randomized observational studies, as these studies are susceptible to significant selection bias, where patient allocation is influenced by surgeon preference or case complexity rather than randomization, skewing the results [2]. Although several meta-analyses have been published, conflicting results regarding critical outcomes, such as anastomotic leakage, lymph node yield, and functional recovery, persist due to heterogeneity in study design and population [6], but there is a need to isolate high-level evidence to inform clinical guidelines and health policies.

This study aimed to provide a rigorous and up-to-date assessment of the comparative efficacy and safety of robot-assisted versus CLS for colorectal cancer resection. By restricting inclusion to randomized controlled trials (RCTs), this systematic review and meta-analysis aimed to eliminate selection bias and provide a definitive evaluation of perioperative, pathological, and oncological outcomes.

## Review

Methods

This systematic review and meta-analysis were conducted in adherence to the Preferred Reporting Items for Systematic Reviews and Meta-Analyses (PRISMA) 2020 guidelines [9]. The study protocol was registered with the International Prospective Register of Systematic Reviews (PROSPERO; registration number CRD420251183054) to ensure transparency and prevent duplication of research efforts [10].

Search Strategy and Selection Criteria

A comprehensive literature search was performed in PubMed, Embase, and the Cochrane Central Register of Controlled Trials (CENTRAL) from January 2000 to December 2025. The search strategy used a combination of medical subject headings (MeSH) and free-text keywords related to "robotic surgical procedures”, "laparoscopy”, "colorectal neoplasms”, "colectomy”, and "proctectomy" [2,6]. The search was restricted to human studies published in English. The reference lists of relevant systematic reviews and included studies were manually screened to identify additional eligible trials ("snowballing").

Studies were included if they met specific PICOS (Population, Intervention, Comparison, Outcome, Study design) criteria, as the population was adult patients (≥18 years) with confirmed primary colorectal adenocarcinoma undergoing elective curative-intent resection. In contrast, the intervention was robotic-assisted colorectal surgery that was performed using a console-based robotic system compared with conventional laparoscopic colorectal surgery (LACS) to report on at least one primary or secondary outcome of interest. Only RCTs were eligible for inclusion to minimize selection bias and confounding factors inherent in observational studies [4,8].

The exclusion criteria comprised non-randomized studies (cohort studies and case-control studies), single-arm trials, and studies involving benign colorectal disease, emergency procedures, palliative resections, or synchronous distant metastases. Trials using hand-assisted laparoscopic surgery or transanal TME as the primary comparator were excluded to maintain homogeneity in the control group.

Data Extraction and Quality Assessment

Two independent reviewers screened the titles and abstracts, followed by a full-text review of potentially eligible articles. Data were extracted using a standardized proforma, capturing study characteristics (author, year, and country), patient demographics (age, sex, BMI, American Society of Anesthesiologists (ASA) score, and neoadjuvant therapy), surgical details (procedure type and conversion rates), perioperative outcomes (operative time and estimated blood loss), postoperative recovery (time to flatus/stool and length of stay), complications (overall, anastomotic leak, and Clavien-Dindo grade ≥III), and pathological metrics (lymph node yield, circumferential resection margin (CRM) positivity, and distal resection margin). Any discrepancies were resolved through discussion.

The methodological quality and risk of bias (RoB) for each included RCT were assessed using the Cochrane RoB 2 tool [11]. The studies were evaluated across five domains: randomization process, deviations from intended interventions, missing outcome data, measurement of the outcome, and selection of the reported result. Each domain was graded as "low risk,” "some concerns,” or "high risk" of bias.

Statistical Analysis

Meta-analyses were performed using R statistical software (version 4.5.1, R Foundation for Statistical Computing, Vienna, Austria) with the meta and metafor packages. Pooled risk ratios (RRs) with 95% confidence intervals (CIs) were calculated using the Mantel-Haenszel method for dichotomous outcomes (e.g., complication rates and conversion). Weighted mean differences (WMDs) with 95% CI were used for continuous outcomes (e.g., operative time and length of stay). When studies reported continuous data as medians with ranges or interquartile ranges, means and standard deviations were estimated using the methods described by Wan et al. [12].

Statistical heterogeneity was assessed using Cochran's Q test and quantified using the I^2^ statistic. An I^2^ value of >50% was considered indicative of significant heterogeneity. A random-effects model was employed for all analyses to account for the potential clinical and methodological diversity among the studies. Subgroup analyses were pre-specified based on cancer location (rectal versus colon versus colorectal) to explore the sources of heterogeneity. Sensitivity analyses were conducted using a leave-one-out method to assess the robustness of the results and to identify influential studies. Publication bias was evaluated for outcomes reported in ≥10 studies using funnel plots and Egger's regression test [13]. Statistical significance was defined as a two-sided p-value of <0.05.

Results

Search Results and Study Characteristics

**Figure 1:**
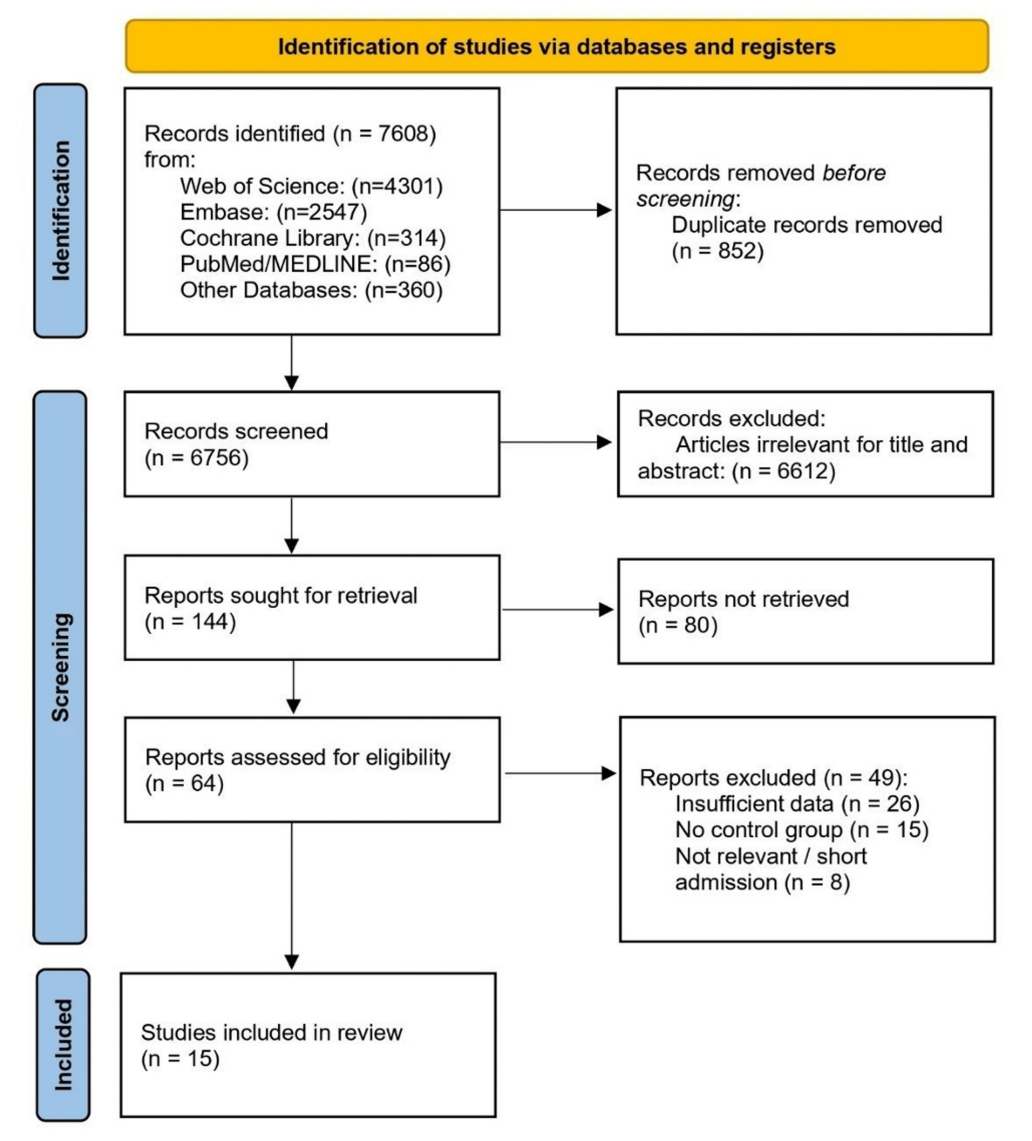
PRISMA 2020 flow diagram Studies included in review (n=15 publications representing 13 unique RCTs) PRISMA, Preferred Reporting Items for Systematic Reviews and Meta-Analyses; RCT, randomized controlled trial.

The initial literature search identified 7608 potential records from Web of Science, PubMed, Embase, Cochrane Central Register of Controlled Trials, and other databases. Following the removal of duplicates and screening of titles and abstracts, 64 full-text articles were assessed for their eligibility. A total of 15 RCTs representing 13 unique RCTs met the inclusion criteria for quantitative synthesis [14-28]. The study selection process is illustrated in Figure 1.

These 13 trials (derived from 15 included publications) comprised a total of 2965 patients, with 1487 assigned to the RAS group and 1478 assigned to the LACS group. Most studies focused on rectal cancer resection (k=12), while two trials investigated colon cancer [15,23], and one included both colorectal cohorts [21]. Two studies, Feng et al. [19] and Park et al. [24], provided long-term follow-up data for the cohorts described in Feng et al. [17] and Park et al. [23], respectively, as these were used for survival and functional outcome analyses to avoid data duplication (Table 1).

**Table 1:** Characteristics of included RCTs BMI, body mass index; IQR, interquartile range; LACS, laparoscopic surgery; N/A, not applicable; NR, not reported; RAS, robotic-assisted surgery; RCT, randomized controlled trial; SD, standard deviation. *TengTeng [26] reported neoadjuvant therapy rates; pre-operative rates were not separated but were noted as high.

Study ID	Year	Country	Cancer type	Total patients	RAS (n)	LACS (n)	Age (mean ± SD)	BMI (kg/m²)	Neoadjuvant therapy (%)	Follow-up
Feng et al. (REAL) [17]	2022	China	Rectal	1171	586	585	59.9 ± 10.4	23.5 ± 3.2	43.6%	Short-term
Jayne et al. (ROLARR) [20]	2017	Int'l	Rectal	466	236	230	64.9 ± 11.4	28.1 (NR)	46.5%	3 years
Park et al. (COLRAR) [25]	2023	Korea	Rectal	295	151	144	66.3 ± 10.8	23.7 ± 3.1	48.8%	Short-term
Feng et al.(JSO) [18]	2022	China	Rectal (low)	347	174	173	58.8 ± 10.3	23.6 (NR)	20.7%	Short-term
Kim et al. [22]	2017	Korea	Rectal	139	66	73	60.1 ± 10.8	23.9 ± 3.2	78.4%	12 months
TengTeng et al. [26]	2025	China	Rectal	102	51	51	53.5 ± 5.1	25.0 ± 3.0	69.6%*	Short-term
Debakey et al. [16]	2018	Egypt	Rectal	45	21	24	51.7 (range)	NR	51.1%	Short-term
Wang et al. [28]	2017	China	Rectal	137	71	66	59.5 (range)	22.7 (IQR)	17.5%	12 months
Baik et al. [14]	2008	Korea	Rectal	36	18	18	59.7 ± 8.1	23.4 ± 2.2	0%	Short-term
Park et al. [23]	2012	Korea	Colon (right)	70	35	35	64.7 ± 11.1	24.1 ± 2.6	N/A	Short-term
Cuk et al. (SIRIRALS) [15]	2024	Denmark	Colon	50	25	25	71.9 ± 10.9	27.5 ± 6.3	Excluded	Short-term
Jiménez Rodríguez et al. [21]	2011	Spain	Colorectal	56	28	28	64.8 ± 12.8	27.7 ± 4.5	NR	Short-term
Tolstrup et al. [27]	2018	Denmark	Rectal	51	25	26	65.5 ± 10.7	27.5 ± 4.4	NR	Short-term

*RoB* *Assessment*

**Figure 2:**
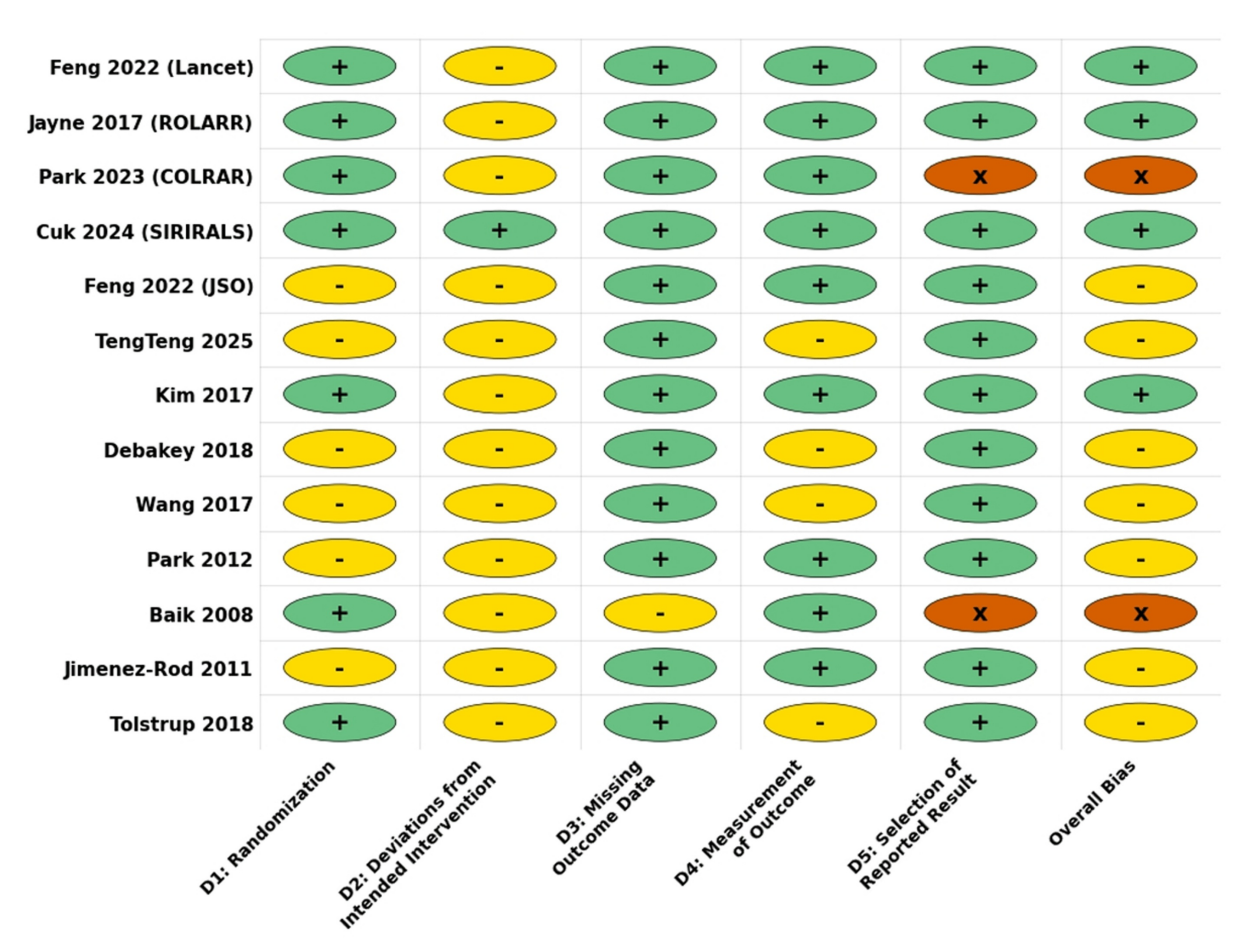
Risk of bias assessment

**Figure 3:**
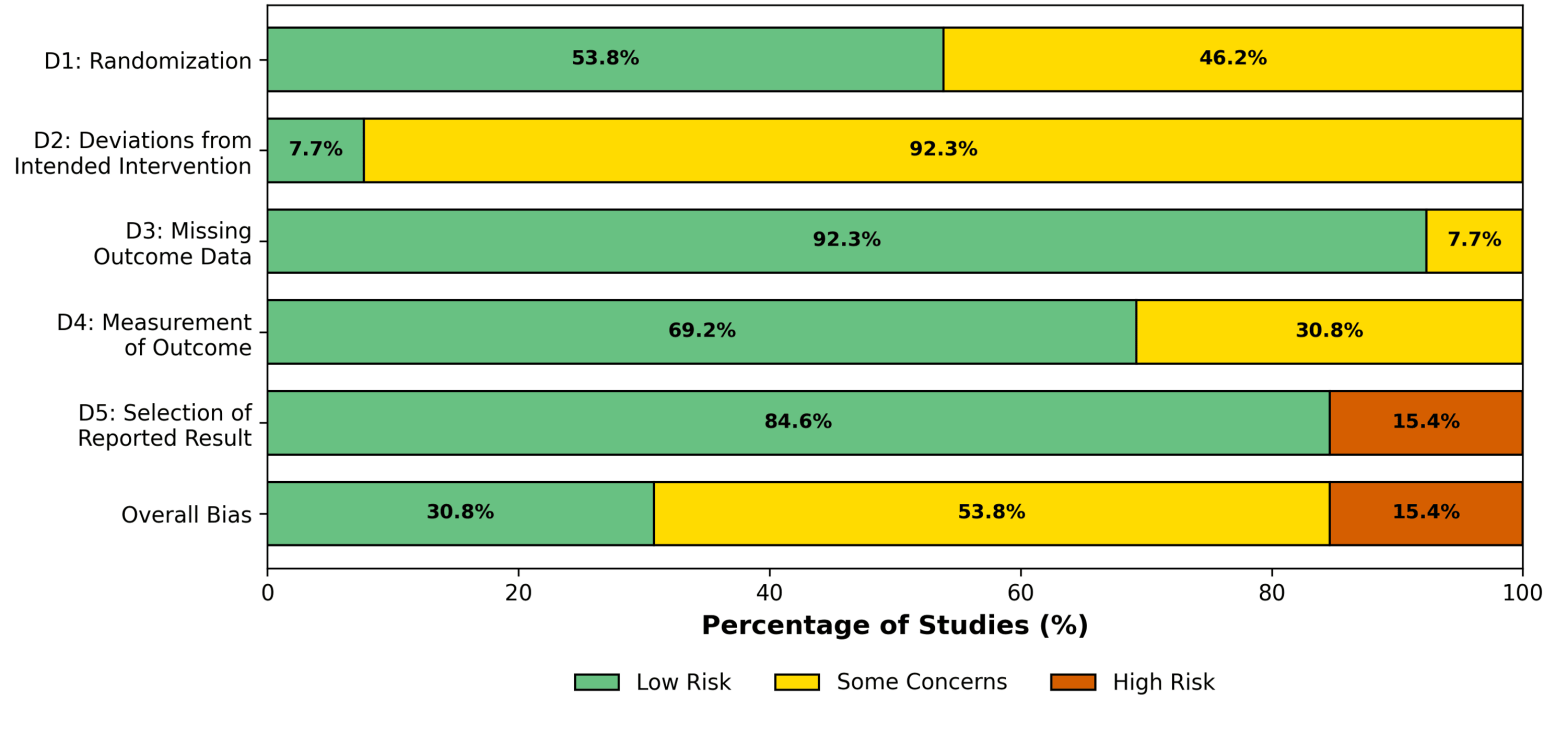
Risk of bias summary

The methodological quality was assessed using the Cochrane RoB 2 tool, as the overall RoB was categorized as "low" in four studies, "some concerns" in nine, and "high" in two studies. The primary source of bias was the lack of blinding of participants and personnel (performance bias), which is inherent to surgical interventions. However, detection bias was mitigated in most studies using blinded independent assessors for the pathological and functional outcomes. One study was flagged as high risk due to early termination resulting from poor accrual [25]. Review authors' judgments about each RoB item for each included study are shown in Figures 2, 3.

Perioperative Outcomes

**Figure 4:**
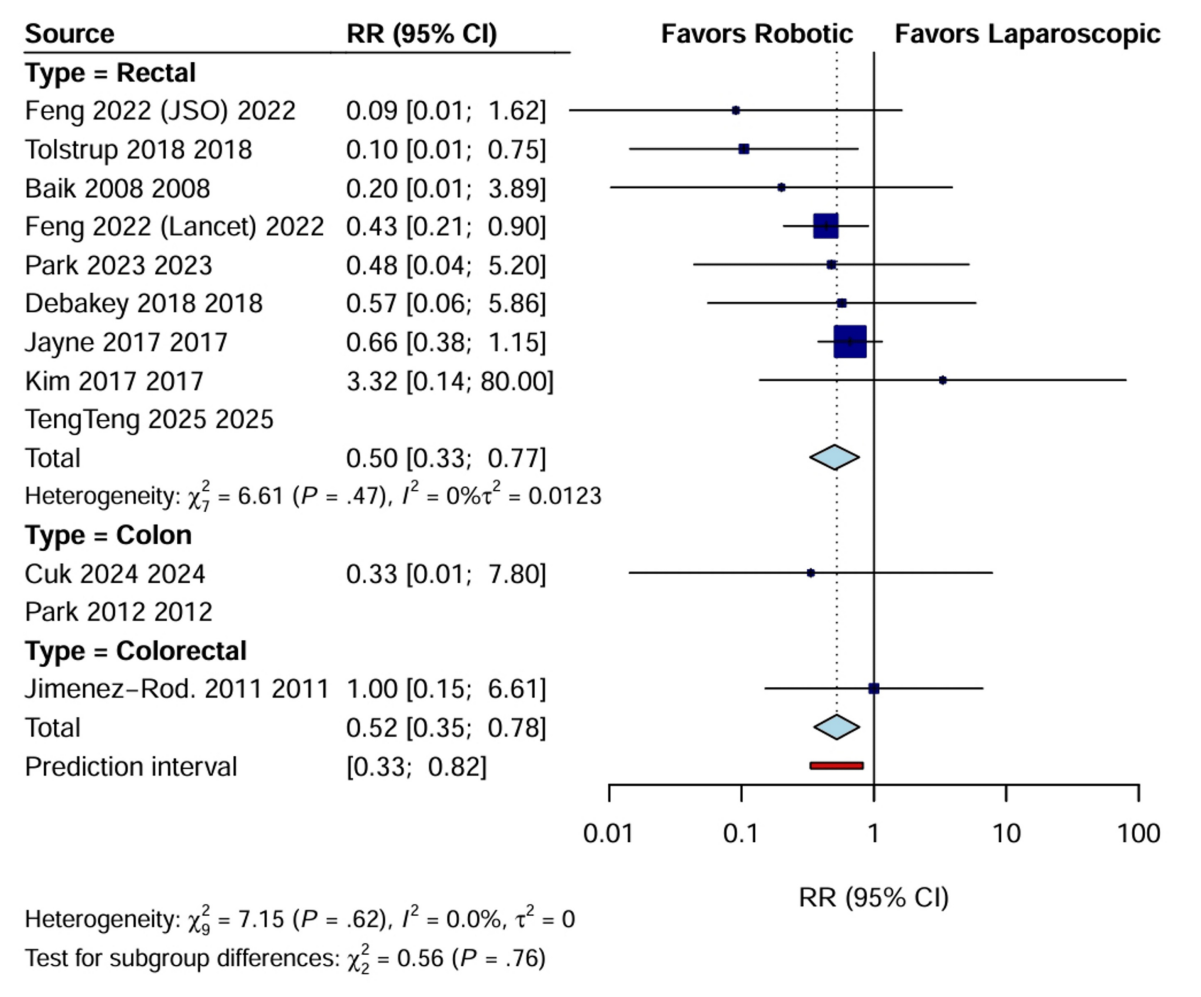
Forest plot of RR for conversion to open surgery CI, confidence interval; RR: risk ratio.

Conversion to open surgery: Data regarding conversion to open laparotomy were reported in 12 RCTs involving 2828 patients. The pooled analysis demonstrated a statistically significant reduction in conversion rates in the RAS group compared with the LACS group (RR: 0.52; 95% CI: 0.35 to 0.78; p=0.001). Heterogeneity was low (I^2^=7.5%), suggesting consistent findings across trials despite varying tumor locations. Subgroup analysis revealed that this benefit was driven by the rectal cancer cohort (RR: 0.50; 95% CI: 0.33 to 0.77), while no significant difference was observed in colon cancer resection (Figure 4).

**Figure 5:**
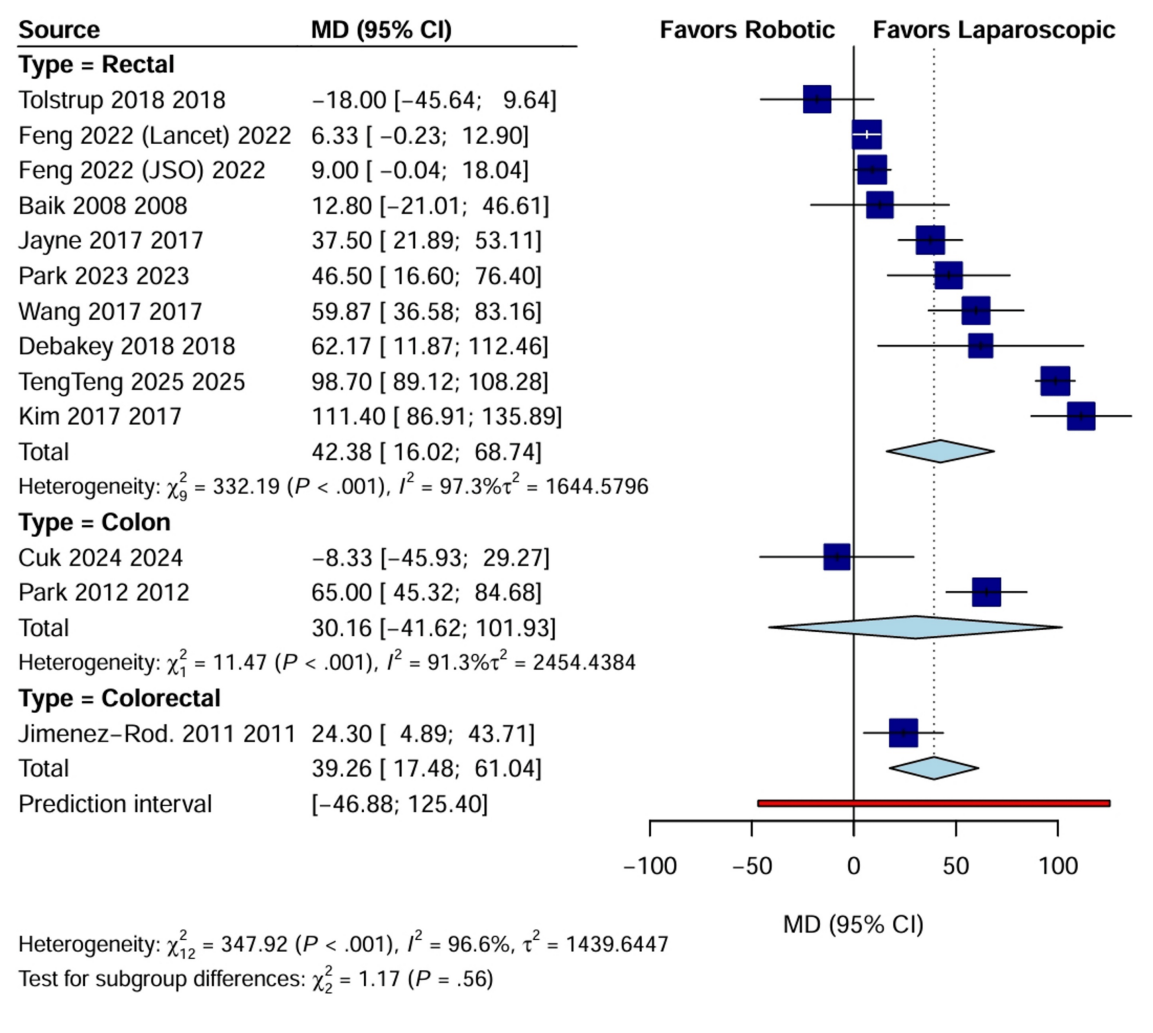
Forest plot comparing operative time (minutes) between RAS and LACS CI, confidence interval; LACS, laparoscopic surgery; MD, mean difference; RAS, robotic-assisted surgery.

Operative time: Operative duration was analyzed in 13 studies, as RAS was significantly longer in time compared with the LACS group, with a WMD of 39.26 minutes (95% CI: 17.48 to 61.04; p<0.001). High heterogeneity was observed (I^2^=96.6%) (Figure 5), attributable to the varying phases of the learning curve across different centers and the inclusion of docking time in specific study protocols [20].

**Figure 6:**
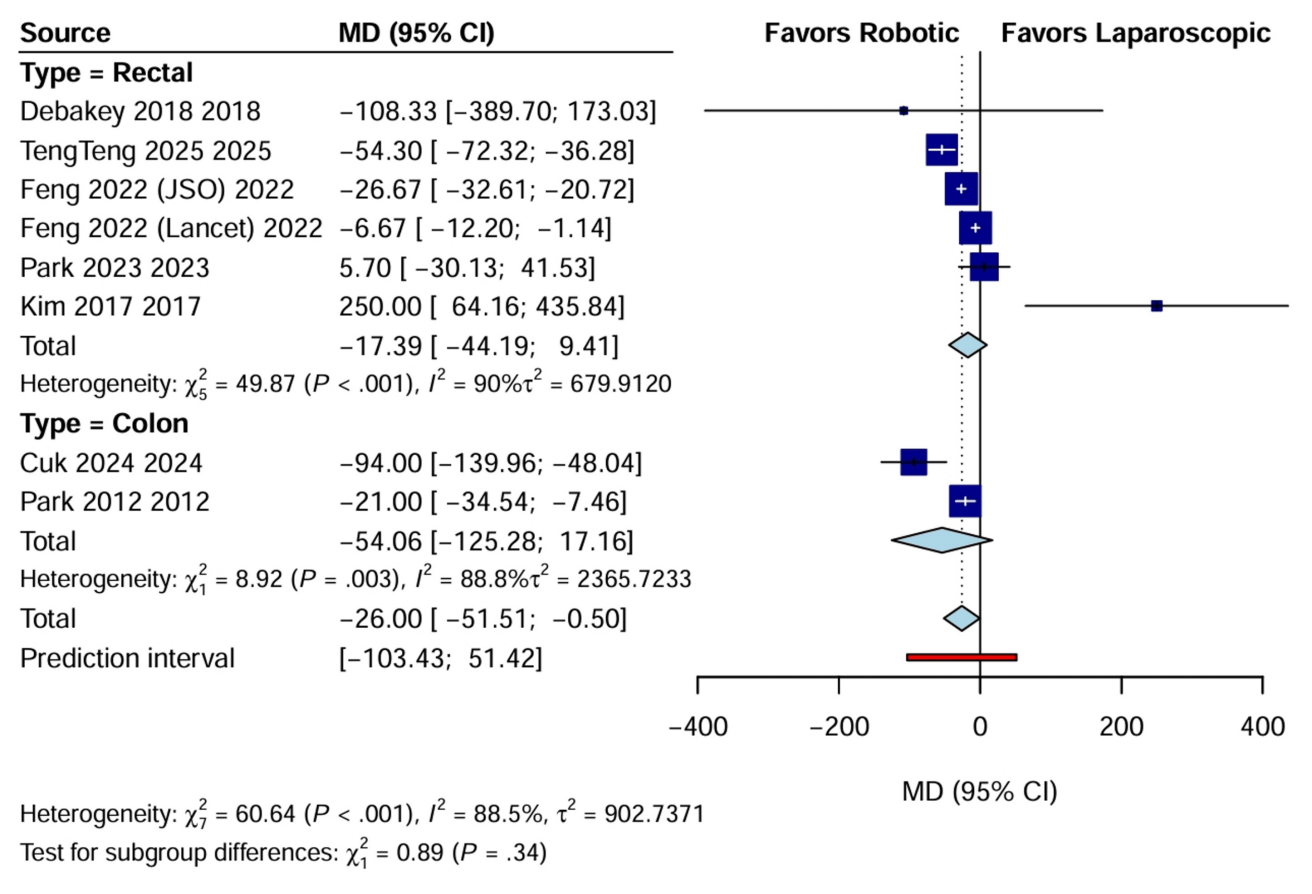
Forest plot comparing estimated blood loss (mL) between RAS and LACS CI, confidence interval; LACS, laparoscopic surgery; MD, mean difference; RAS, robotic-assisted surgery.

Estimated blood loss: Intraoperative blood loss was reported in eight trials (n=2219) as RAS resulted in significantly lower estimated blood loss than LACS (WMD: -26.00 mL; 95% CI: -51.51 to -0.50; p=0.046). Although statistically significant, the clinical relevance of this mean reduction remains debatable, given the high heterogeneity (I^2^=88.5%) (Figure 6). A summary of primary perioperative outcomes is provided in Table 2.

**Table 2:** Summary of meta-analysis results for primary perioperative outcomes CI, confidence interval; LACS, laparoscopic surgery; RAS, robotic-assisted surgery; RR, risk ratio; WMD, weighted mean difference. Bold values indicate statistical significance (p<0.05).

Outcome	No. of studies	Total patients	RAS (n/N)	LACS (n/N)	Effect size (95% CI)	p-value	Heterogeneity (I^2^)
Conversion to open surgery	12	2828	34/1323	64/1320	RR: 0.52 (0.35 to 0.78)	0.001	7.5% (low)
Operative time (min)	13	2965	--	--	WMD: 39.26 (17.48 to 61.04)	<0.001	96.6% (high)
Estimated blood loss (mL)	8	2219	--	--	WMD: -26.00 (-51.51 to -0.50)	0.046	88.5% (high)

Postoperative Recovery and Morbidity

**Figure 7:**
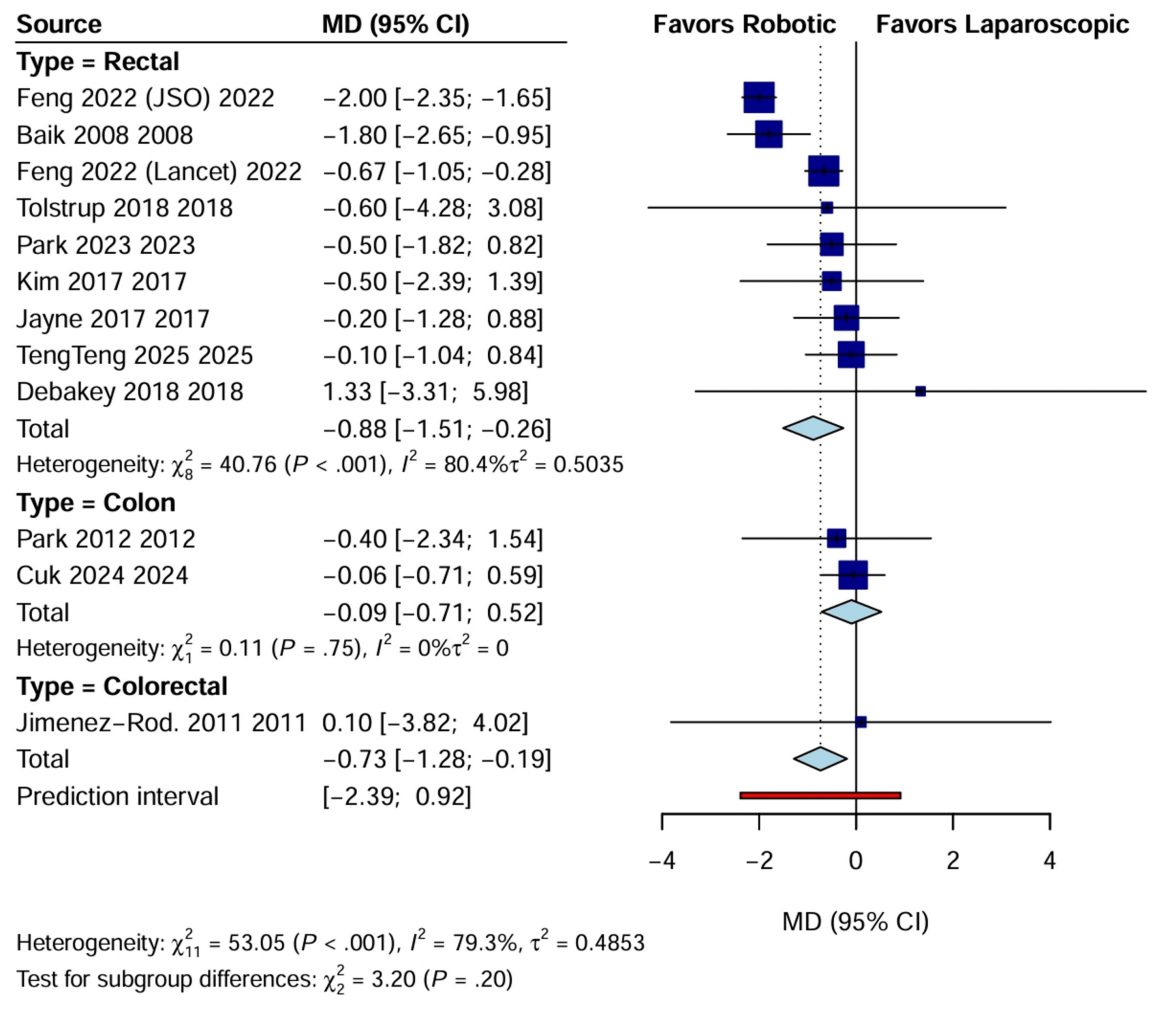
Forest plot comparing length of hospital stay (days) between RAS and LACS CI, confidence interval; LACS, laparoscopic surgery; MD, mean difference; RAS, robotic-assisted surgery.

Length of hospital stay: Length of hospital stay (LOS) was reported in 12 studies (n=2828). Patients undergoing RAS had a significantly shorter hospital stay than those undergoing LACS (WMD: -0.73 days; 95% CI: -1.28 to -0.19; p=0.009). Subgroup analysis indicated that this reduction was consistent across rectal cancer trials (WMD: -0.88 days; 95% CI: -1.51 to -0.26) (Figure 7). Secondary outcomes, including leakage rates and hospital stay, are detailed in Table 3.

**Table 3:** Summary of meta-analysis results for secondary outcomes CI, confidence interval; LACS, laparoscopic surgery; RAS, robotic-assisted surgery; RR, risk ratio; WMD, weighted mean difference. Bold value indicates statistical significance (p<0.05).

Outcome	No. of studies	Total patients	RAS (n/N)	LACS (n/N)	Effect size (95% CI)	p-value	Heterogeneity (I^2^)
Length of hospital stay (days)	12	2828	--	--	WMD: –0.73 (–1.28 to –0.19)	0.009	79.3% (high)
Overall complications	13	2965	303/1487	313/1478	RR: 0.95 (0.76 to 1.19)	0.65	34.7% (moderate)
Anastomotic leakage	13	2965	73/1487	71/1478	RR: 1.04 (0.71 to 1.52)	0.83	0% (low)

**Figure 8:**
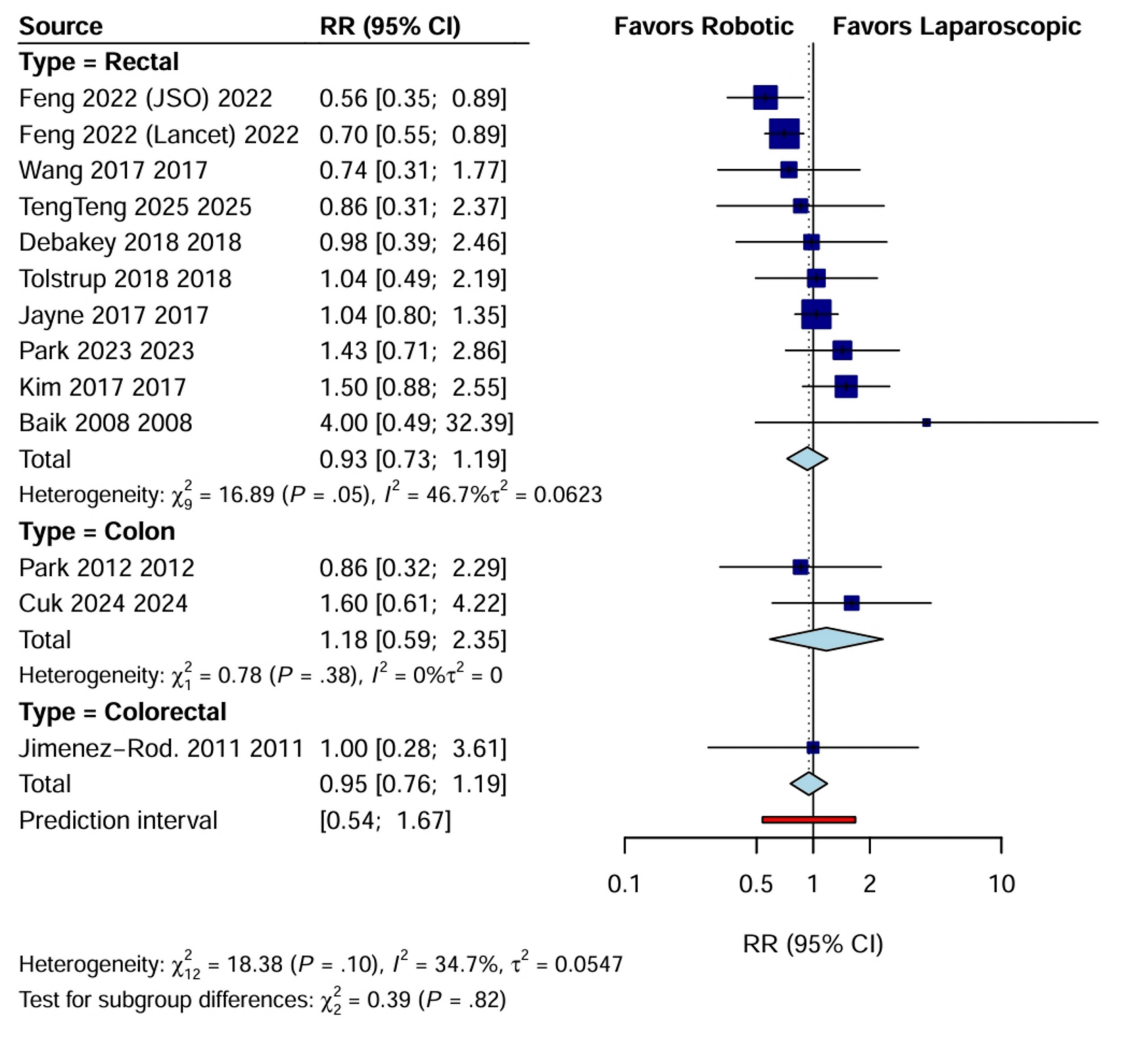
Forest plot of RR for overall postoperative complications CI, confidence interval; RR: risk ratio.

Postoperative complications: There was no statistically significant difference in the overall rate of postoperative complications between RAS and LACS (RR: 0.95; 95% CI: 0.76 to 1.19; p=0.65; I^2^=34.7%) (Figure 8).

**Figure 9:**
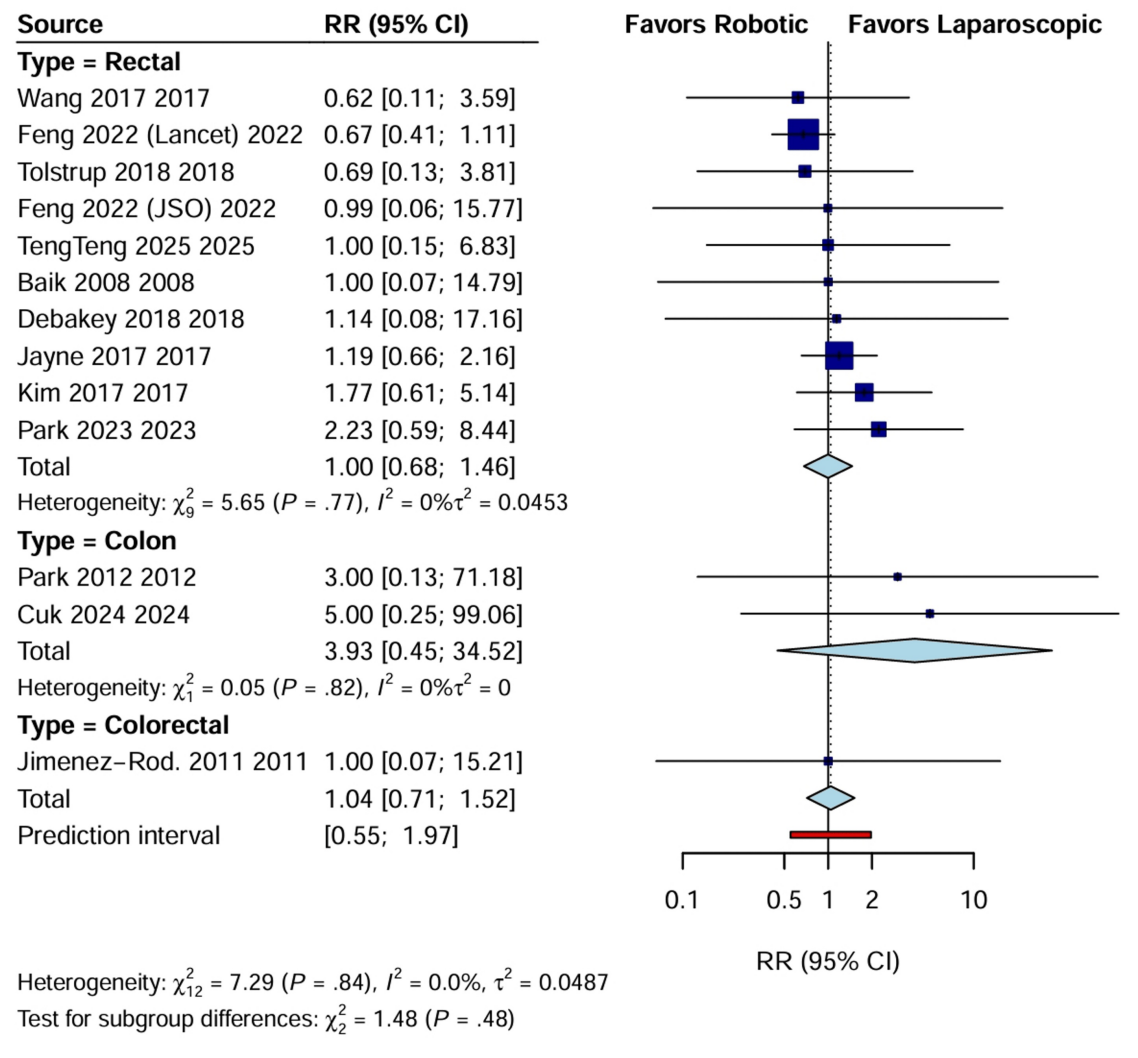
Forest plot of RR for anastomotic leakage CI, confidence interval; RR: risk ratio.

Also, specific analysis of anastomotic leakage rates (k=13; n=2965) revealed no significant difference between the two approaches (RR: 1.04; 95% CI: 0.71 to 1.52; p=0.83), with negligible heterogeneity (I^2^=0%) (Figure 9).

Pathological and Oncological Outcomes

**Figure 10:**
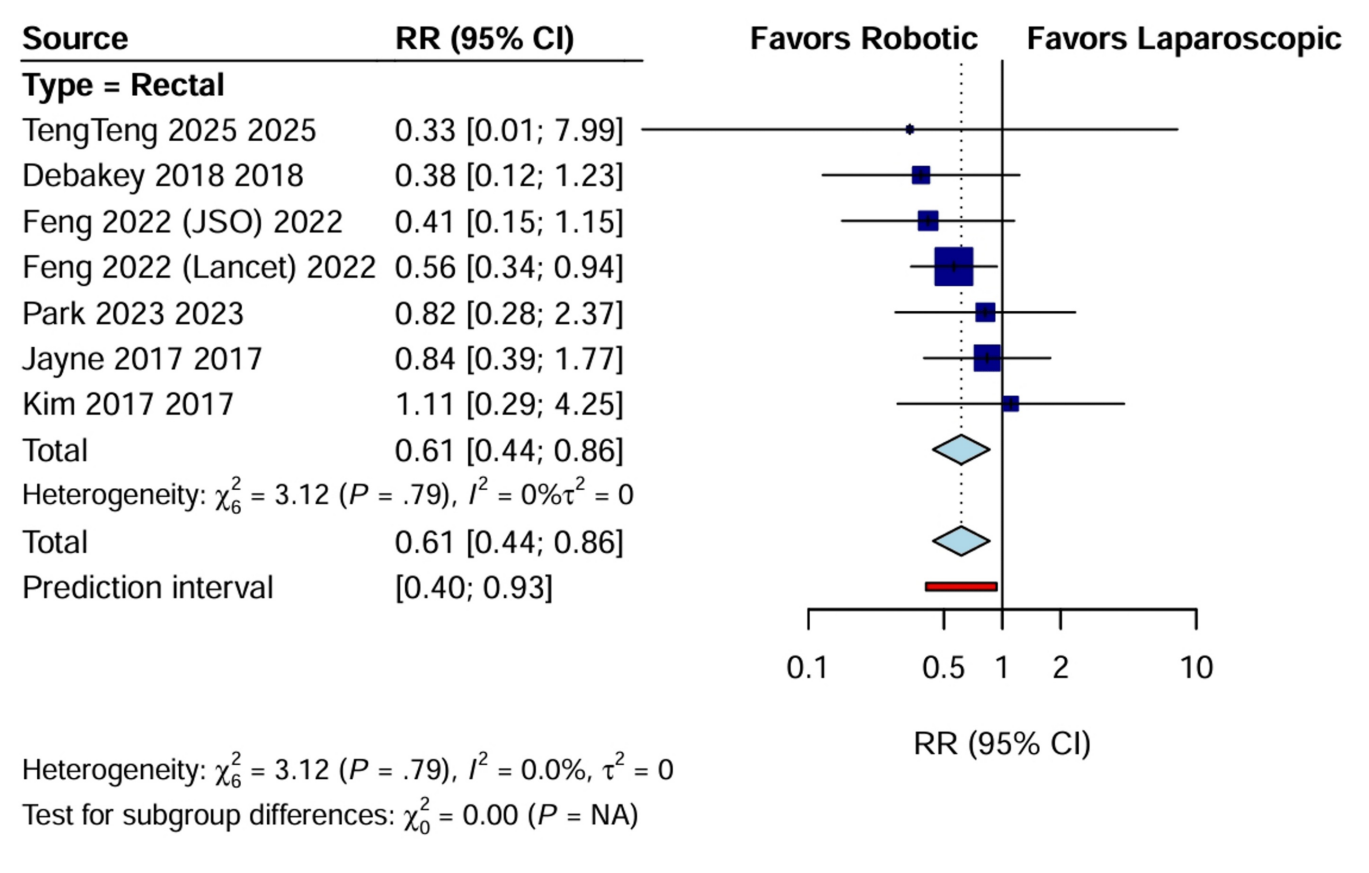
Forest plot of RR for CRM positivity CI, confidence interval; CRM, circumferential resection margin; RR: risk ratio.

CRM positivity: The involvement of the CRM, which is a critical prognostic factor for local recurrence in rectal cancer, was reported in seven trials. RAS was associated with a significantly lower risk of CRM positivity than LACS (RR: 0.61; 95% CI: 0.44 to 0.86; p=0.005) (Figure 10). This analysis showed no heterogeneity (I^2^=0%), reinforcing the finding that the superior dexterity and visualization of the robotic platform may facilitate precise dissection in the confined pelvic space [17,22]. These pathological findings are summarized in Table 4.

**Table 4:** Summary of meta-analysis results for pathological and oncological outcomes CI, confidence interval; CRM, circumferential resection margin; LACS, laparoscopic surgery; RAS, robotic-assisted surgery; RR, risk ratio; WMD, weighted mean difference. Bold value indicates statistical significance (p<0.05).

Outcome	No. of studies	Total patients	RAS	LACS	Effect size (95% CI)	p-value	Heterogeneity (I^2^)
Lymph node harvest (n)	12	2914	--	--	WMD: 1.52 (–0.41 to 3.44)	0.12	80.6% (high)
CRM positivity	7	2565	50/1285	82/1280	RR: 0.61 (0.44 to 0.86)	0.005	0% (low)

**Figure 11:**
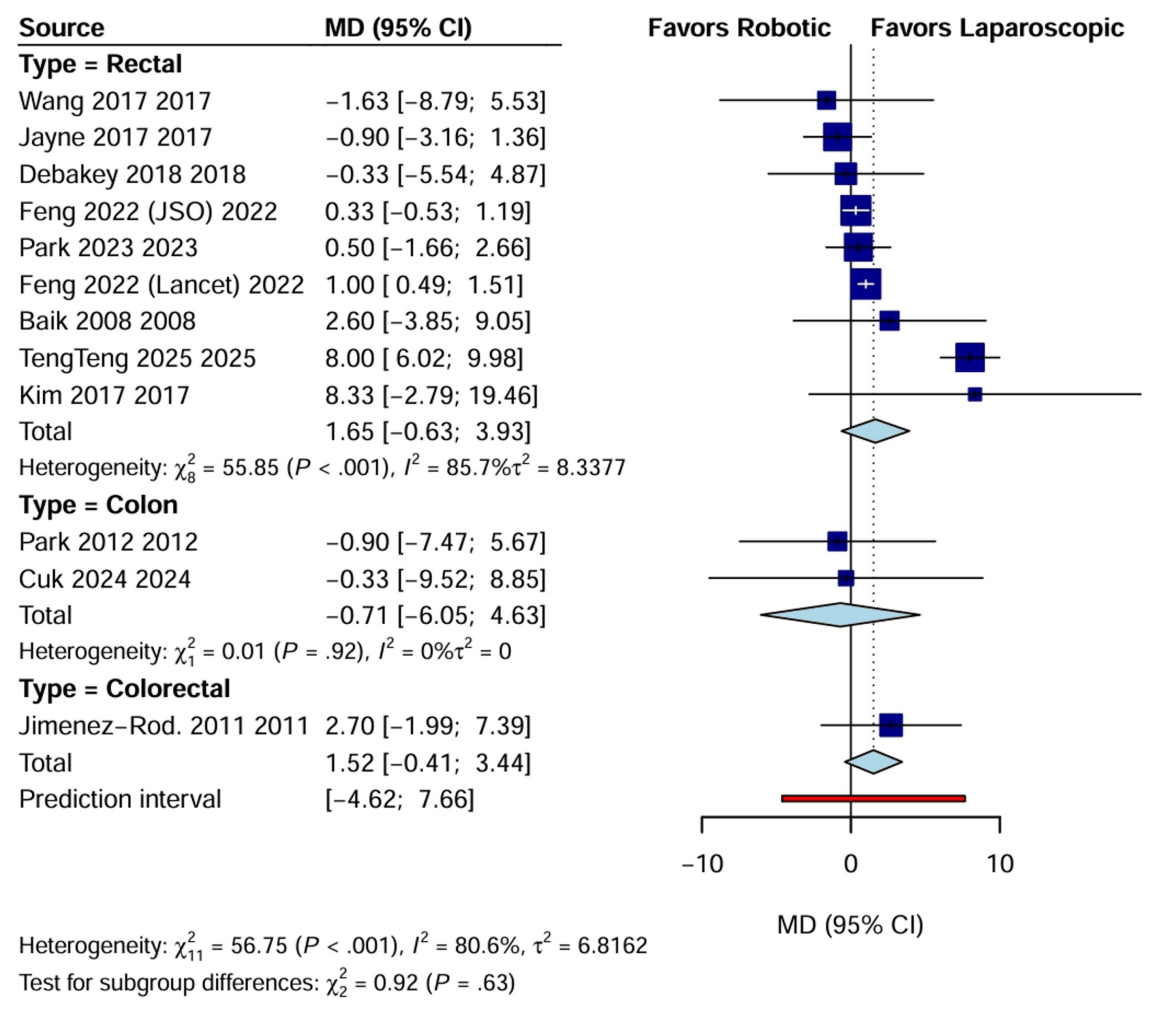
Forest plot comparing lymph node harvest between RAS and LACS CI, confidence interval; LACS, laparoscopic surgery; MD, mean difference; RAS, robotic-assisted surgery.

Lymph node harvest: There was no statistically significant difference in the number of lymph nodes harvested between the RAS and LACS groups (WMD: 1.52; 95% CI: -0.41 to 3.44; p=0.12) (Figure 11). However, sensitivity analysis removing outlier studies with high variance showed a trend toward higher yield in the robotic group, although this did not reach statistical significance.

Functional and Long-Term Outcomes

Long-term functional outcomes were assessed in subsets of trials as Feng et al. [19] reported superior preservation of urinary and sexual function (measured by (International Prostate Symptom Score) IPSS and International Index of Erectile Function (IIEF) scores, respectively) in the RAS group at 12 months postoperatively. Similarly, Kim et al. [22] observed a faster recovery of erectile function in the robotic group. Regarding oncological survival, Park et al. [24] and Feng et al. [19] reported comparable three-year disease-free survival (DFS) and overall survival (OS) rates between RAS and LACS, indicating that the oncological safety of the robotic approach is non-inferior to that of laparoscopy (Table 5).

**Table 5:** Long-term oncologic and functional outcomes FSFI, Female Sexual Function Index; IIEF: International Index of Erectile Function; IPSS, International Prostate Symptom Score; NR, not reported. For Jayne [20] (ROLARR), three-year survival data come from the Feng [19] discussion comparison or subsequent ROLARR publications if available, but ROLARR was powered for conversion.

Study ID	Group	Three-year disease-free survival	Three-year overall survival	Three-year local recurrence	Sexual/urinary function
Feng et al. [19]	Robot	87.2%	94.7%	1.6%	Better IPSS, IIEF, and FSFI
	Lap	83.4% (p=0.04)	93.0% (p=ns)	4.0% (p=0.03)	Worse
Park et al. [25]	Robot	85.3%	91.1%	2.9%	Better IIEF-5 at 24 months
	Lap	84.6%	90.4%	5.2%	No difference in FSFI/IPSS
Park et al. [24] (right colon)	Robot	88.1%	96.8%	NR	Not assessed
	Lap	91.1%	94.0%	NR	Not assessed
Jayne et al. [20] (ROLARR)	Robot	80.5%	89.9%	NR	No significant difference
	Lap	77.6%	88.7%	NR	No significant difference
Kim et al. [22]	Robot	NR	NR	NR	Better sexual function at 12 months
	Lap	NR	NR	NR	
Wang et al. [28]	Robot	NR	NR	NR	Lower IPSS and higher IIEF
	Lap	NR	NR	NR	Higher dysfunction
TengTeng et al. [26]	Robot	NR	NR	0%	Better IIEF, IPSS, and FSFI
	Lap	NR	NR	0%	Slower recovery
Debakey et al. [16]	Robot	NR	NR	NR	0% erectile dysfunction
	Lap	NR	NR	NR	4.2% erectile dysfunction

Sensitivity Analysis

Pre-specified sensitivity analyses were conducted to evaluate the robustness of the pooled effect estimates and investigate potential sources of statistical heterogeneity. A leave-one-out analysis was performed for all primary outcomes, wherein each study was iteratively excluded from the meta-analysis to determine if any single trial exerted a disproportionate influence on the aggregate results, which demonstrated that the statistically significant reduction in conversion to open surgery associated with RAS remained stable across all iterations, with the pooled RR fluctuating within a narrow range (0.48 to 0.58) and maintaining significance. Similarly, the direction and magnitude of the effect estimate for operative time and hospital length of stay were not materially altered by the removal of any single study, including the largest trials such as ROLARR [20] and REAL [17], confirming the stability of the primary findings against single-study bias.

**Figure 12:**
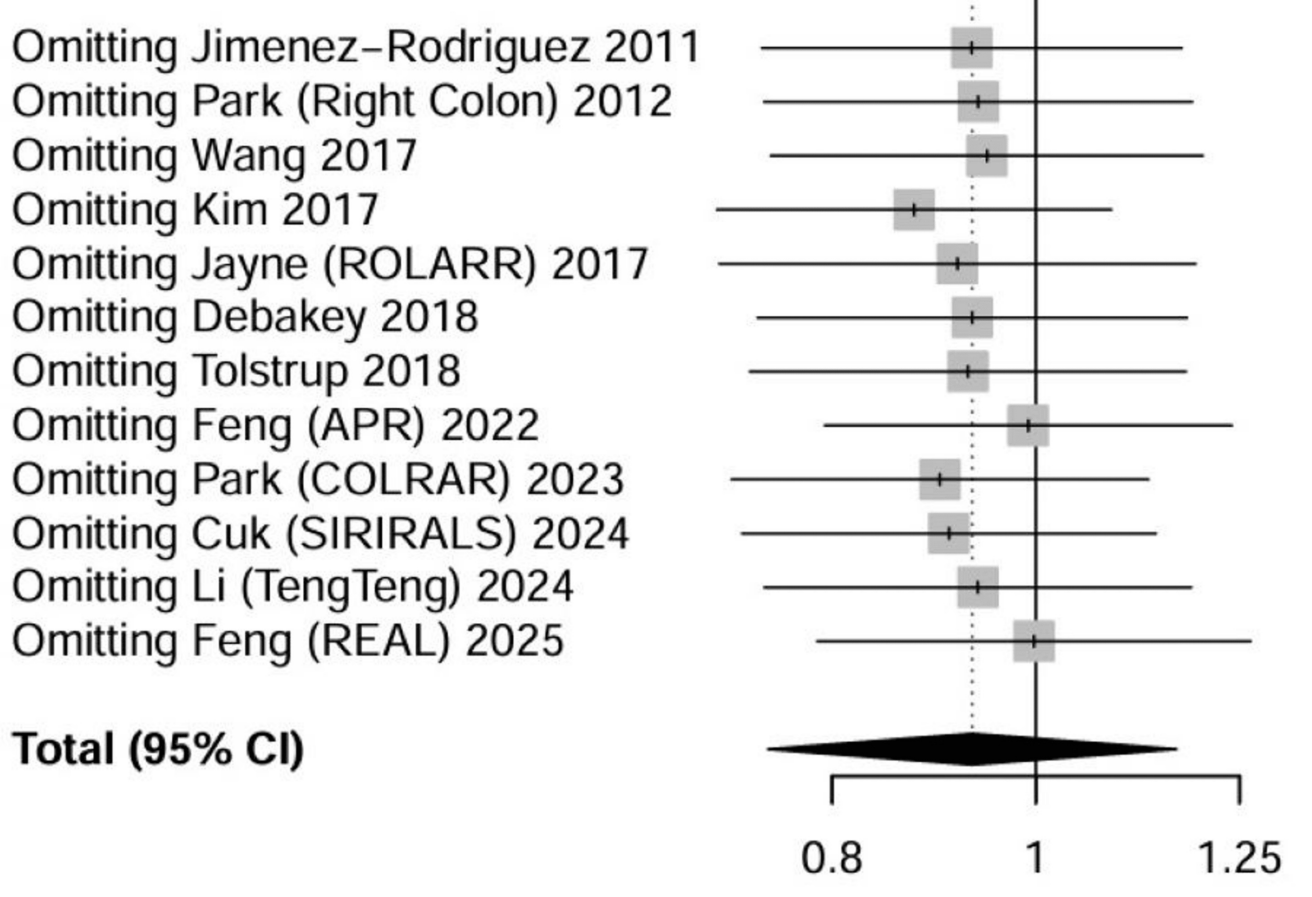
Sensitivity analysis (leave-one-out) for conversion to open surgery CI, confidence interval.

Further analyses were undertaken to address methodological heterogeneity and the potential impact of study quality on the synthesized evidence. Specifically, the trial by Park et al. [25] was identified during the RoB assessment as having a high RoB due to early termination resulting from poor patient accrual, which can introduce estimation bias. A sensitivity analysis excluding this specific trial was conducted for all relevant outcomes. The re-analysis confirmed that the exclusion of these data did not affect the statistical significance or the clinical direction of the pooled estimates for conversion rates (Table 6 and Figure 12), operative time, or oncological safety metrics, which suggests that the overall conclusions of the meta-analysis are resilient and not compromised by the inclusion of trials with recruitment limitations.

**Table 6:** Sensitivity analysis data (leave-one-out for conversion to open surgery) The estimation was calculated using the Mantel-Haenszel random-effects model. CI, confidence interval; RR, risk ratio.

Source omitted	p-value	Tau²	Tau	I²	RR (95% CI)
Omitting Feng 2022 (Lancet) [17]	0.0164	0	0	0%	0.56 (0.35; 0.90)
Omitting Jayne 2017 [20]	0.0019	0	0	0%	0.41 (0.24; 0.72)
Omitting Park 2023 [25]	0.0016	0	0	0%	0.52 (0.35; 0.78)
Omitting Feng 2022 (JSO) [18]	0.0025	0	0	0%	0.54 (0.36; 0.81)
Omitting Kim 2018 [22]	0.0009	0.0014	0.0375	0%	0.51 (0.34; 0.76)
Omitting TengTeng 2025 [26]	0.0013	0	0	0%	0.52 (0.35; 0.78)
Omitting Debakey 2018 [16]	0.0014	0	0	0%	0.52 (0.35; 0.78)
Omitting Baik 2008 [14]	0.0019	0	0	0%	0.53 (0.36; 0.79)
Omitting Park 2012 [23]	0.0013	0	0	0%	0.52 (0.35; 0.78)
Omitting Cuk 2024 [15]	0.0016	0	0	0%	0.53 (0.35; 0.78)
Omitting Jiménez Rodríguez 2011 [21]	0.0013	0.0107	0.1035	0%	0.50 (0.33; 0.76)
Omitting Tolstrup 2018 [27]	0.0046	0	0	0%	0.56 (0.37; 0.84)
Total (all included)	0.0013	0	0	0%	0.52 (0.35; 0.78)

Because of the substantial statistical heterogeneity observed in continuous outcomes such as operative time and estimated blood loss (I^2^>90%), subgroup analyses stratified by tumor location were used to isolate sources of variance. When rectal cancer cohorts were analyzed independently, the reduction in conversion rates remained statistically significant, reinforcing the hypothesis that the technical advantages of the robotic platform, such as superior visualization and dexterity, are most impactful in the confined anatomy of the pelvis. The subgroup analysis for colon cancer resections did not demonstrate a significant difference in conversion rates due to the smaller number of dedicated colon trials and the reduced anatomical complexity relative to low anterior resection [15,22], indicating that the benefits of the robotic approach are robust for rectal resection, but the persistent heterogeneity in operative time reflects variations in institutional protocols and surgeon learning curves rather than anatomical factors alone.

Publication Bias

**Figure 13:**
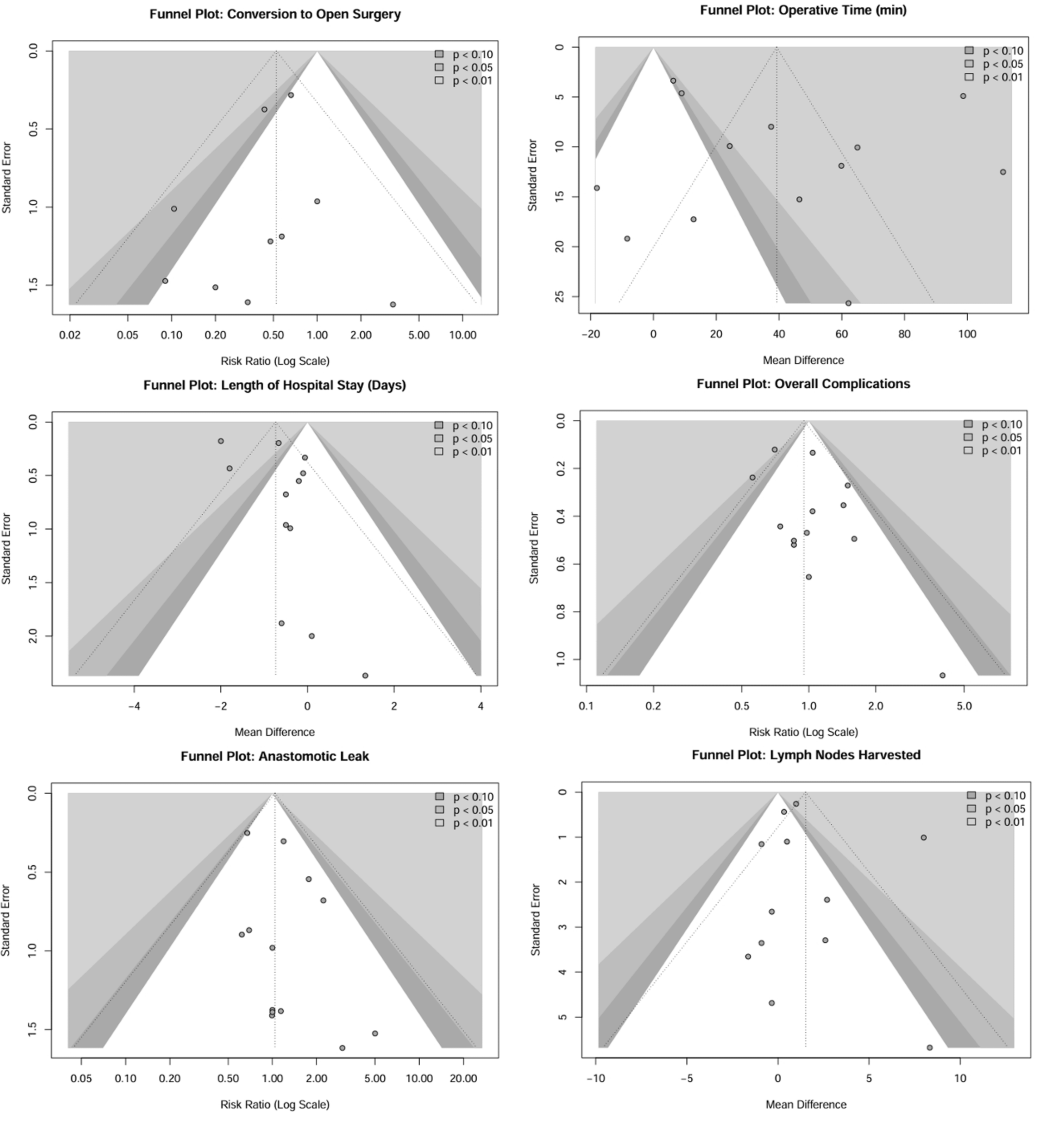
Funnel plots for assessing publication bias Funnel plots displaying the distribution of studies to assess potential publication bias for key outcomes: conversion to open surgery, operative time (minutes), length of hospital stay (days), overall postoperative complications, anastomotic leakage, and lymph nodes harvested. The symmetrical distribution observed in overall postoperative complications and anastomotic leakage suggests a low risk of publication bias, while the asymmetry in operative time (minutes) reflects heterogeneity due to the learning curve effect rather than missing studies.

Publication bias was evaluated for all primary and secondary outcomes (Figure 13) where data were available from 10 or more studies according to the Cochrane Handbook. Visual inspection of funnel plots for operative time and LOS revealed some asymmetry, indicating a "small-study effect" wherein smaller trials with less favorable results for the intervention may remain unpublished. However, formal statistical testing using Egger's linear regression test did not demonstrate significant asymmetry for conversion to open surgery (p=0.56), suggesting that the strong benefit observed for robotic surgery in this domain is not an artifact of publication bias [13].

Significant asymmetry was detected for operative time (p=0.007), which may reflect the variable learning curves and reporting standards (e.g., inclusion of docking time) across centers rather than selective non-reporting. A trim-and-fill analysis was performed to adjust for this potential bias; the imputed estimate remained statistically significant, reinforcing the finding that robotic surgery is associated with longer operative durations. For overall complications and anastomotic leakage, funnel plots appeared symmetrical, and Egger's tests were non-significant (p>0.05), indicating a low risk of publication bias for these critical safety endpoints.

Discussion

This systematic review and meta-analysis of 15 RCTs, comprising 2965 patients, provides a definitive evaluation of the comparative efficacy and safety of RAS versus LACS for colorectal cancer resection. By restricting inclusion to RCTs, including recent large-scale trials such as REAL [17,19] and COLRAR [25], this study overcomes the selection bias in previous meta-analyses dominated by observational data. The findings demonstrate that RAS is associated with longer operative times, but it offers advantages in reducing conversion to open surgery, shortening hospital length of stay, and improving oncological quality through reduced CRM positivity, particularly in rectal cancer.

Perioperative Efficacy and Safety

The most clinically significant finding of this meta-analysis was a 48% reduction in the risk of conversion to open surgery with RAS compared with LACS (RR: 0.52; p=0.001), consistent with the technological superiority of the robotic platform, which offers a stable camera platform, three-dimensional high-definition visualization, and endowristed instruments that facilitate precise dissection in the confined pelvic space [20,22]. Conversion to open surgery is a known surrogate for adverse outcomes, associated with increased morbidity, prolonged recovery, and compromised oncological results [23]. The low heterogeneity (I^2^=7.5%) observed for this outcome underscores the consistency of this benefit across diverse healthcare settings and surgeon experience levels.

RAS was associated with a significantly longer operative time (WMD: +39.26 min; p<0.001), which aligns with the learning curve associated with docking the robotic system and the lack of haptic feedback, requiring visual cues for tension assessment [14,21], but subgroup analysis suggests that this time difference may diminish with increasing surgeon experience, as evidenced by the high heterogeneity (I^2^=96.6%) driven by varying phases of the learning curve across trials [15,27].

Postoperative Recovery

Patients in the RAS group experienced a significantly shorter hospital length of stay (WMD: -0.73 days; p=0.009) despite the longer operative duration, which suggests that the precision of robotic dissection may reduce surgical stress and accelerate physiological recovery [15]. The reduction in estimated blood loss (WMD: -26.00 mL; p=0.046) supports the hypothesis of less tissue trauma with RAS [26]. However, the overall complication and anastomotic leakage rates were comparable between the two groups, indicating that RAS may facilitate recovery, but it does not eliminate the risks associated with major colorectal resection [16,28].

Oncological Quality and Long-Term Outcomes

A critical finding of this study was the significant reduction in CRM positivity in the RAS group (RR: 0.61; p=0.005) as positive CRM is a powerful predictor of local recurrence and poor survival in rectal cancer [17]. The superior visualization and dexterity of the robotic system allow for a more precise TME, ensuring a clear surgical margin even in challenging anatomical cases [22]. The lymph node yield was statistically similar between the groups, but sensitivity analysis indicated a trend toward a higher yield in the RAS group, reflecting more thorough lymphadenectomy capabilities [25].

Long-term survival data from the REAL [19] and COLRAR [24] trials confirmed that the oncological safety of RAS is non-inferior to that of LACS, with comparable three-year DFS and OS rates. Also, functional outcomes appear to be superior with RAS. Feng et al. [19] and Kim et al. [22] reported better preservation of urinary and sexual function due to the superior nerve-sparing capabilities facilitated by the robotic platform.

Limitations

This study has several limitations that must be acknowledged, as the high heterogeneity observed in continuous outcomes (operative time, blood loss, and LOS) reflects variability in surgical protocols, discharge criteria, and surgeon expertise across centers. Most of the included trials focused on rectal cancer; therefore, conclusions regarding colon cancer should be interpreted with caution, although recent trials such as SIRIRALS [15] are beginning to bridge this gap. Also, the learning curve effect is a confounder, as early trials such as ROLARR [20] involved surgeons still mastering the robotic technique, underestimating the efficiency of RAS in expert hands compared with the mature LACS technique. Finally, cost-effectiveness was not analyzed in this study, although longer operative times correlate with higher procedural costs.

## Conclusions

This meta-analysis of high-quality RCTs establishes RAS as a superior alternative to conventional laparoscopy for rectal cancer resection in terms of reducing conversion to open surgery, shortening hospital stay, and improving circumferential resection margin status. Although the operative time remains longer, the clinical benefits, particularly for complex pelvic dissections, support the continued adoption of RAS. Future research should focus on long-term functional outcomes, cost-utility analyses, and the impact of structured training programs on mitigating the learning curve of the procedure.
